# Supplementary material for: Aberrant cortical reorganization and astrogliosis underlie memory deficit post-traumatic brain injury: a longitudinal multiscale multimodal study
Source: bioRxiv. 2026 Oct 22:2026.10.16.759571. Preprint. [Version 1] doi: 10.64898/2026.10.16.759571 (PMC13738506; doi:10.64898/2026.10.16.759571)
Supplement: Supplementary file 1 [file NIHPP2026.10.16.759571v1-supplement-1.pdf]

bioRxiv preprint doi: https://doi.org/10.64898/2026.10.16.759571; this version posted October 22, 2026. The copyright holder for this preprint (which was not certified by peer review) is the author/funder, who has granted bioRxiv a license to display the preprint in perpetuity. It is made available under a CC-BY 4.0 International license.

SUPPLEMENTARY MATERIAL

Figures S1-S4 and supplementary table 1

bioRxiv preprint doi: https://doi.org/10.64898/2026.10.16.759571; this version posted October 22, 2026. The copyright holder for this preprint (which was not certified by peer review) is the author/funder, who has granted bioRxiv a license to display the preprint in perpetuity. It is made available under a CC-BY 4.0 International license.

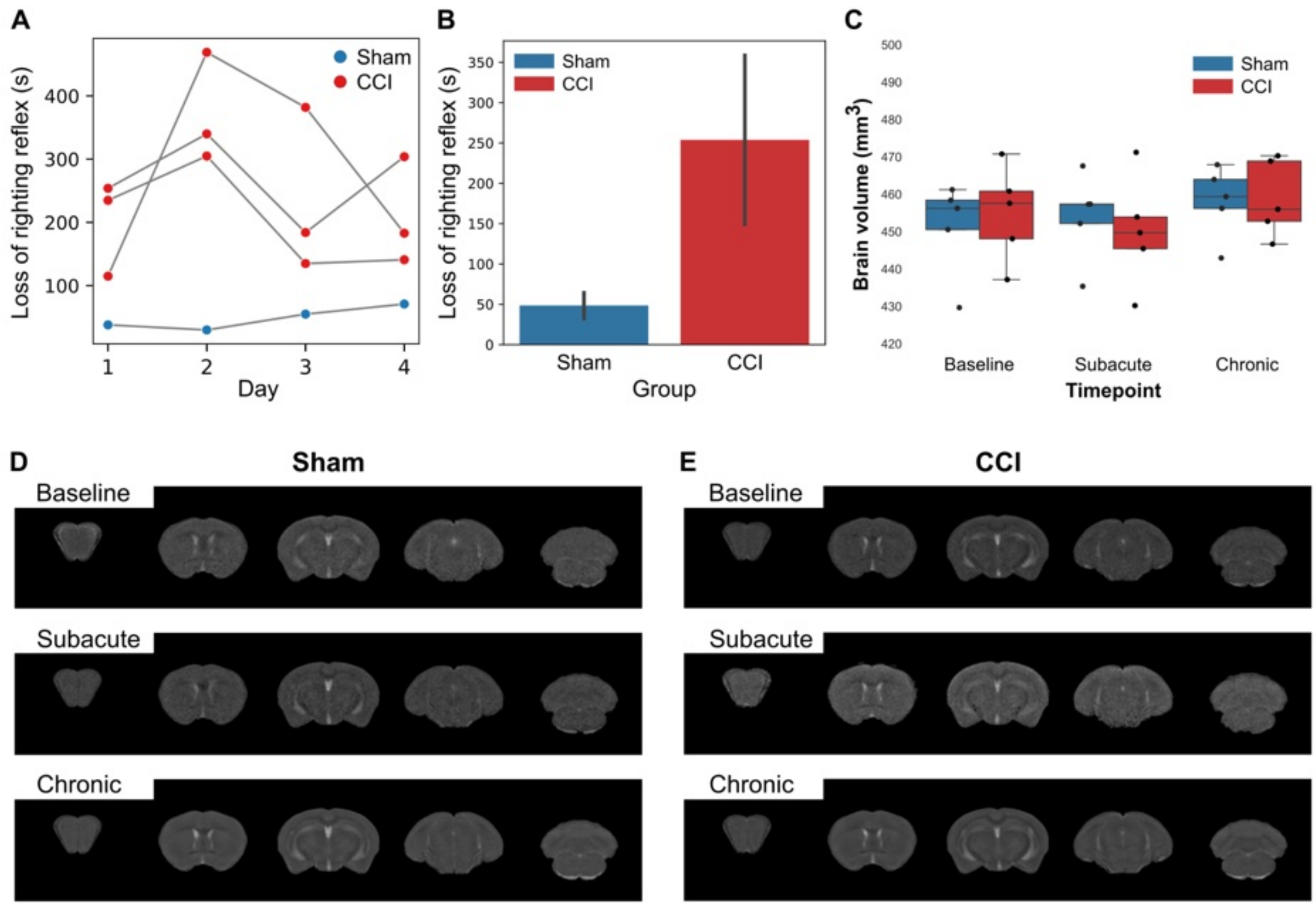

Supplementary Figure 1: Gross sequelae of the CCI model. Characterisation of controlled cortical impact (CCI) severity (A) Duration of loss of righting reflex (LRR) of 1 sham and 3 CCI mice in CCI severity characterisation test on each day of hit. (B) Across all hits, CCI mice had a longer LRR (t-test, t = 3.66, $p < 0.01$). (C) Brain volume (mm3) was estimated from T2 structural scans, which showed no significant difference in brain volume between groups at any timepoints. Representative T2 structural scans of a sham (D) and a CCI (E) mouse, demonstrating absence of contrast.

bioRxiv preprint doi: https://doi.org/10.64898/2026.10.16.759571; this version posted October 22, 2026. The copyright holder for this preprint (which was not certified by peer review) is the author/funder, who has granted bioRxiv a license to display the preprint in perpetuity. It is made available under a CC-BY 4.0 International license.

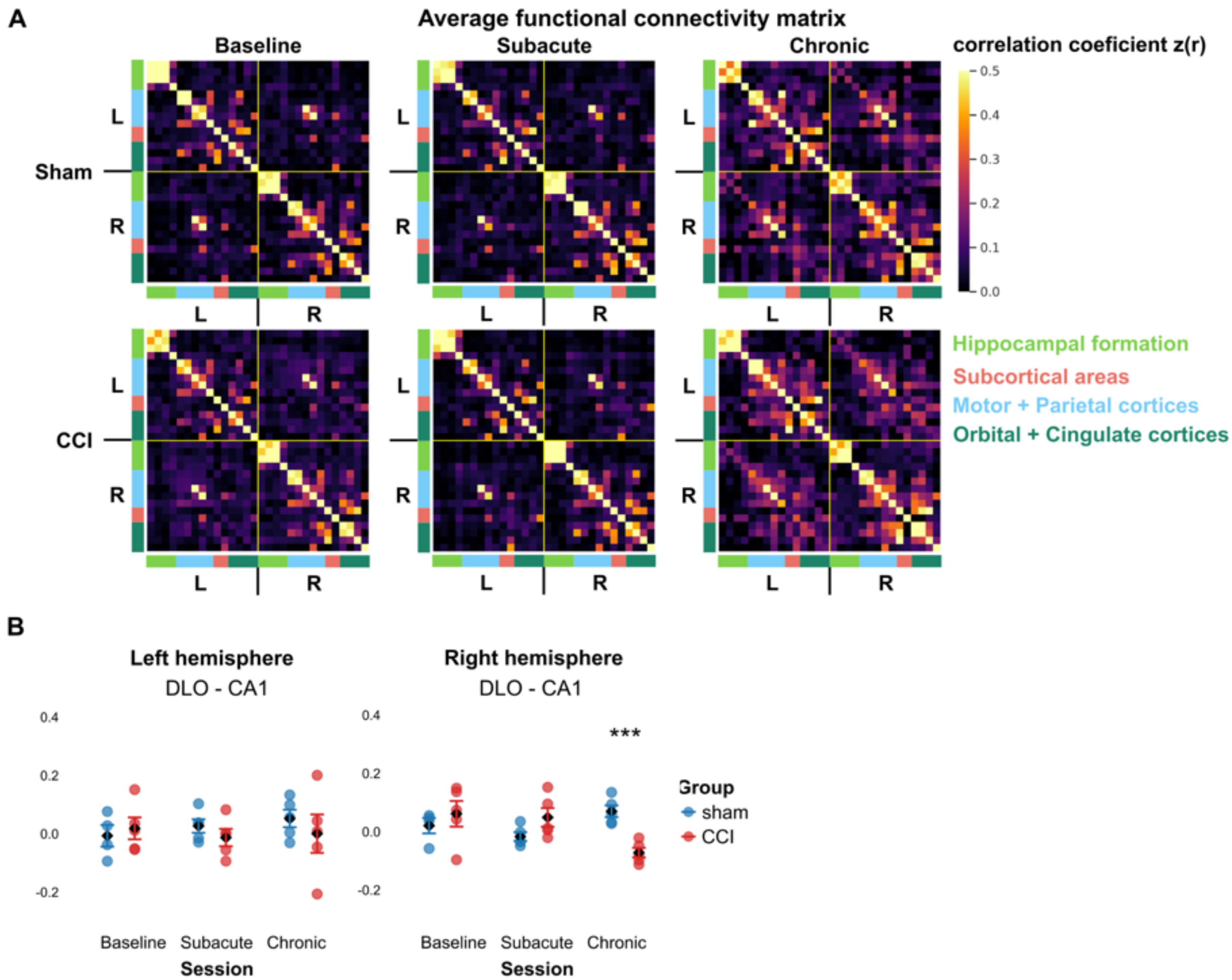

Supplementary Figure 2: Resting-state fMRI results. (A) Groupwise averaged z-scored correlation heatmaps at each timepoint. Color shows group-averaged z-scored correlation. (B) ipsi-contusional DLO-CA1 connectivity was decreased significantly in the CCI group at chronic timepoint. Points represent individual animals colored by group, diamonds indicate group means and error bars represent standard error. CCI: controlled cortical impact; DLO: dorsolateral orbital cortex. Asterisks indicate *p*-value, where $p < 0.05$ *, $p < 0.01$ **, $p < 0.001$ ***.

bioRxiv preprint doi: https://doi.org/10.64898/2026.10.16.759571; this version posted October 22, 2026. The copyright holder for this preprint (which was not certified by peer review) is the author/funder, who has granted bioRxiv a license to display the preprint in perpetuity. It is made available under a CC-BY 4.0 International license.

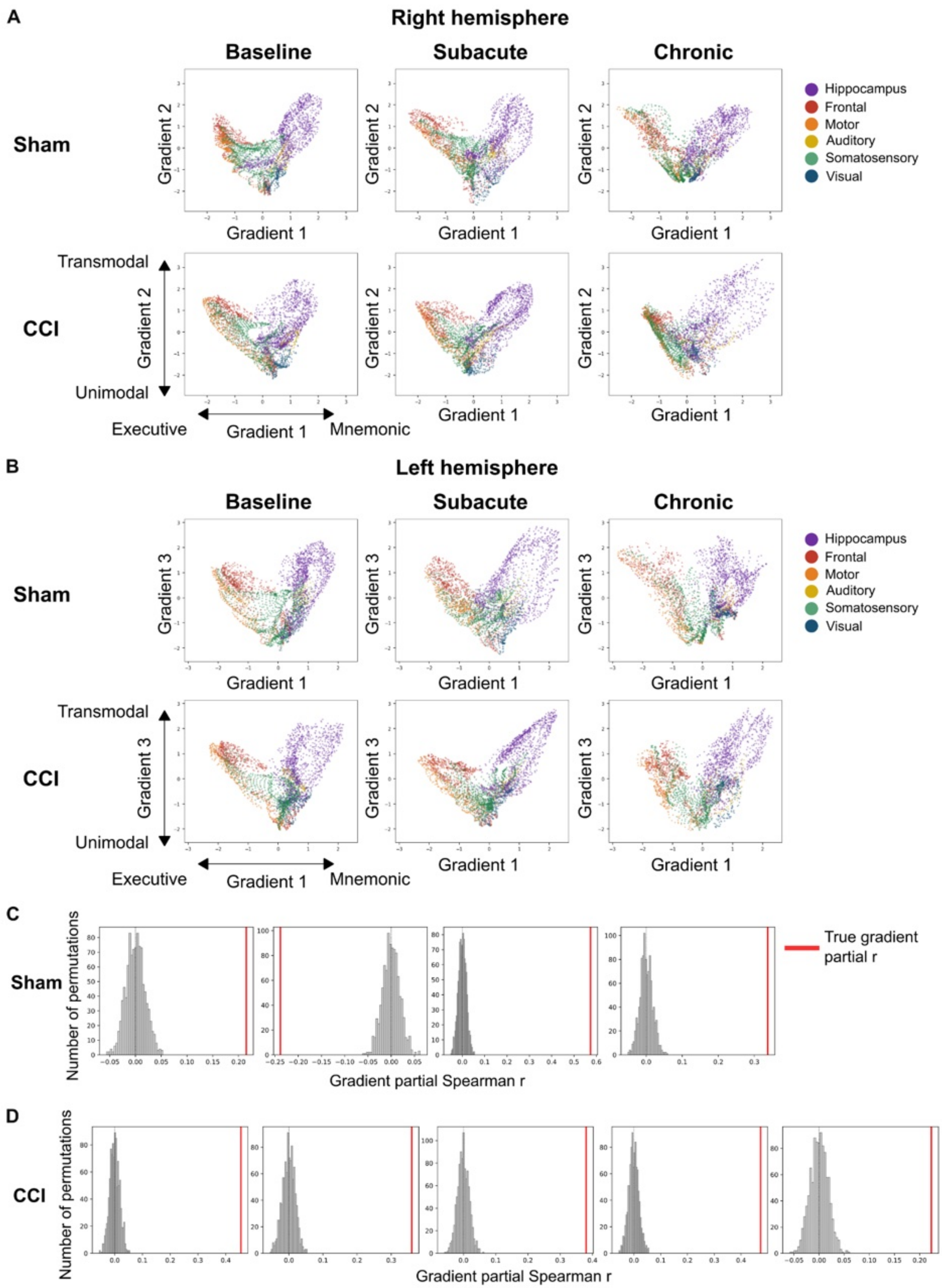

bioRxiv preprint doi: https://doi.org/10.64898/2026.10.16.759571; this version posted October 22, 2026. The copyright holder for this preprint (which was not certified by peer review) is the author/funder, who has granted bioRxiv a license to display the preprint in perpetuity. It is made available under a CC-BY 4.0 International license.

Supplementary Figure 3: Functional connectomic gradient results. Group and timepoint averaged voxelwise gradients in gradient space defined by the executive - mnemonic and transmodal - unimodal gradients for the right ipsi-contusional (A) and left contra-contusional (B) hemispheres. Each point represents a voxel in the fMRI, colored by the functional network it belongs to. (C) Voxel-level permutation confirms spatial correspondence between gradient distance and concordance. For each animal, concordance values were randomly permuted across voxels 1,000 times and the gradient–concordance partial correlation recomputed on each permutation (grey histograms; null mean ≈ 0). Red lines mark the true partial r. Each plot represents a single animal. In three of four sham animals (shown in (C)), the true partial r exceeded all 1,000 permuted values (Fisher combined $p = 1.7 \times 10^{-6}$) while this was true for all CCI animals (shown in (D)) (Fisher combined $p = 6.6 \times 10^{-11}$). This confirmed that the gradient–concordance relationship arose from the true spatial ordering of concordance values rather than an artifact of metric construction, and validated that the selective suppression of anatomical distance as a co-predictor of functional connectivity changes was a CCI-specific effect.

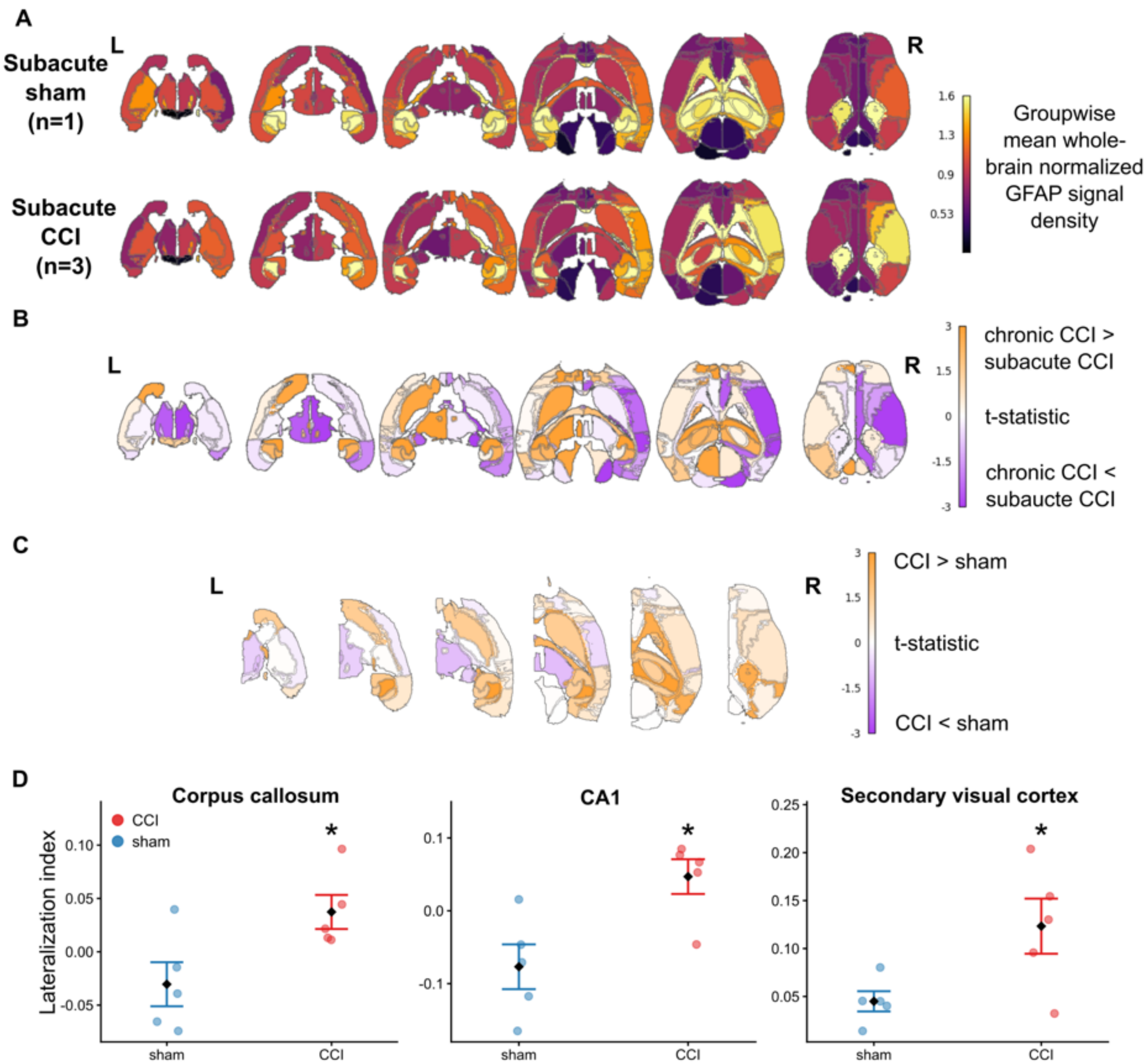

bioRxiv preprint doi: https://doi.org/10.64898/2026.10.16.759571; this version posted October 22, 2026. The copyright holder for this preprint (which was not certified by peer review) is the author/funder, who has granted bioRxiv a license to display the preprint in perpetuity. It is made available under a CC-BY 4.0 International license.

Supplementary Figure 4: GFAP light-sheet fluorescence microscopy results. GFAP signal density elevation in subacute CCI, and increase of lateralization index of GFAP signal density in chronic CCI. (A) Group-averaged whole-brain normalized GFAP signal density maps show an increase in the subacute CCI group compared to the sham. Color scale shows group-averaged whole-brain normalized signal density from segmented GFAP+ astrocytes masks. (B) T-statistic from region-wise groupwise comparisons of LI. Color scale shows t-statistic. Regions in orange indicate higher whole-brain normalized GFAP signal in chronic CCI than subacute CCI while regions in purple indicate lower whole-brain normalized GFAP signal in chronic CCI than subacute CCI. (C) T-statistic from region-wise groupwise comparisons of LI at chronic stage. Color scale shows t-statistic. Regions in orange indicate higher LI in CCI than shams while regions in purple indicate lower LI in CCI than shams. (D) LI was increased in the corpus callosum, CA1 and secondary visual cortex in the CCI group. Points represent individual animals colored by group, diamonds indicate group means and error bars represent standard error. CCI: controlled cortical impact; LI: lateralization index. Asterisks indicate *p*-value, where $p < 0.05$ *, $p < 0.01$ **, $p < 0.001$ ***.

Supplementary Table 1 - Region name abbreviations

Abbreviation	DSURQE atlas region name
CA1	CA10r, LMol, CA1Rad, CA1Py
CA2/CA3	CA2Py, CA20r, CA2Rad, CA3Py Inner, CA3Py Outer, CA30r, CA3Rad, SLu
DG	Dentate gyrus (MoDG, GrDG, PoDG)
EC	Entorhinal cortex (Caudomedial entorhinal cortex, Dorsal intermediate entorhinal cortex, Medial entorhinal cortex, Ventral intermediate entorhinal cortex)
DLO	Dorsolateral orbital cortex
LO	Lateral orbital cortex
MO	Medial orbital cortex
Cg	Cingulate cortex (areas 24a, 24a’, 24b, 24b’, 25, 29a, 29b, 29c, 30, 32)
FrA	Frontal association cortex

bioRxiv preprint doi: https://doi.org/10.64898/2026.10.16.759571; this version posted October 22, 2026. The copyright holder for this preprint (which was not certified by peer review) is the author/funder, who has granted bioRxiv a license to display the preprint in perpetuity. It is made available under a CC-BY 4.0 International license.

Str	Striatum (striatum, olfactory tubercle, fundus of striatum, nucleus accumbens)
Th	Thalamus
MOp	Primary motor cortex
SSp	Primary somatosensory cortex (barrel field, dysgranular zone, forelimb region, hindlimb region, jaw region, shoulder region, trunk region, upper lip region)
VISp	Primary visual cortex
mPPC	Medial parietal association cortex
